# Whisky and Sunstroke

**Published:** 1881-11

**Authors:** 


### WHISKY AND SUNSTROKE.

Dr. Allison Maxwell observes, in the *American Practitioner*, that, while an interne in the Cincinnati Hospital, there were many cases of insolation brought to that institution, and it was a patent fact that most of the cases occurred in drinking men, and that the prognosis in drinkers was unfavorable. He also mentions the fact that in India, on the same day, Sir Charles Napier and forty-three other Europeans were prostrated from heat apoplexy. The forty-three died, and Sir Charles recovered, and explained his recovery by declaring that the sun found no ally of alcohol in his brain.

This, we believe is the experience of every military surgeon in that country, and it is a noteworthy fact that among thousands of mariners who annually visit Calcutta, a proportionately much larger number of captains die from heat apoplexy than common sailors, although the latter are much more exposed, frequently working in the sun all day, while the former usually remain indoors, in cool apartments, or under double awnings.

This can only be explained by the fact that while sailors go on an occasional spree, the captains regale themselves constantly with brandy and soda, in order to "neutralize the injurious effects of the climate."

